# Targeting IL-1β in the Treatment of Atherosclerosis

**DOI:** 10.3389/fimmu.2020.589654

**Published:** 2020-12-10

**Authors:** Wuqian Mai, Yuhua Liao

**Affiliations:** ^1^ Department of Cardiology, Union Hospital, Tongji Medical College, Huazhong University of Science and Technology, Wuhan, China; ^2^ Key Lab of Molecular Biological Targeted Therapies of the Ministry of Education, Union Hospital, Tongji Medical College, Huazhong University of Science and Technology, Wuhan, China

**Keywords:** IL-1β, atherosclerosis, clinical trials, immune system, inflammation, therapy

## Abstract

The role of inflammation in atherosclerosis has been recognized several decades ago and existing treatments provide benefits in part through non-specific anti-inflammatory actions. Compared with other cytokines, interleukin-1β (IL-1β) is associated with acute and chronic inflammation. Anti-inflammatory therapy with canakinumab targeting the IL-1β innate immunity pathway could significantly reduce the rate of recurrent cardiovascular events than placebo. The results of CANTOS suggested an important role of IL-1β in atherosclerosis. However, there are numerous mechanisms that are to be clarified. We herein discussed the important immunomodulatory effect IL-1β exerts on atherosclerosis and the potential mechanisms underlying it. We also reviewed bench-to-bedside clinical translation of IL-1β neutralizing strategies associated with the use of IL-1β blockade in patients with atherosclerosis.

## Introduction

The formation of atherosclerosis is affected by many risk factors. Among them, elevated levels of plasma LDL-cholesterol (LDL-C) is known to be a major one (1–3) However, even when LDL-C was reduced to a sufficiently low level by a PCSK-9 inhibitor, considerable residual cardiovascular risk persists (4–5). In recent years, there are increasing evidences suggesting that atherosclerosis is an active process akin to the chronic inflammatory process and that inflammation was present at all stages of atherosclerosis (6, 7). Lipid and inflammation are related to each other. The accumulation and oxidation of LDL-C at intimae will promote inflammation and drive atherosclerosis (8). Immune cell activation will also decrease cholesterol efflux and foam cell formation, thus influencing progression of atherosclerosis and emerging of complications (9, 10). Therefore, it is critical to break the network of inflammation during atherosclerosis. A large clinical trial, Canakinumab Anti-inflammatory Thrombosis Outcome Study (CANTOS) has proved the effectiveness of anti-inflammatory therapy in treating atherosclerosis (11) and demonstrated the important role of interleukin-1β (IL-1β) in promoting atherosclerosis. In light of the satisfying result produced by CANTOS, we aim to clarify the role of interleukin-1 (IL-1) and related signaling pathways in atherosclerosis. In addition, we reviewed anti-inflammatory treatment strategies that involved targeting IL-1 in animal and clinical research. In this review, we will discuss the choice of IL-1β or interleukin-1α (IL-1α) as a target and proper markers in clinical trials.

## The Role of IL-1β in Atherosclerosis

Plenty of evidence has shown the important effect IL-1β exerts on atherosclerosis (12, 13). First of all, the protein and mRNA levels of IL-1β in atherosclerosis patients increased significantly compared with normal subjects; the levels are also positively correlated with the severity of the disease (14, 15). In addition, the variation of IL-1 gene families is also related to coronary heart disease (CHD) (16–18). Moreover, the increased susceptibility of atherosclerosis associated with the presence of clonal hematopoiesis in peripheral-blood cells was regulated at least in part by the NLRP3/IL-1β pathway (19, 20).

### Synthesis of IL-1β

IL-1β is synthesized predominantly by monocytes, macrophages and dendritic cells (21), and the synthesis of IL-1β can be divided into several steps. At first, IL-1β precursor (pro-IL-1β) and NLRP3 were transcribed, translated and synthesized. This happens when there is a pro-inflammatory signal that activate pattern recognition receptors (PRR), or when IL-1α and IL-1β bind to IL-1 receptor(IL-1R) to form a positive feedback loop (9, 21). The pro-inflammatory signals include cholesterol crystals or oxidized low density lipoprotein(ox-LDL) accumulated under the intimae. Next, NLRP3 is activated by cholesterol crystals engulfed by macrophages, ox-LDL or efflux of potassium. Activated NLRP3 then recruits the adaptor protein, ASC (apoptosis-associated speck-like protein containing a caspase recruitment domain [CARD]) to ligate NLRP3 and pro-caspase-1, facilitating the latter to be hydrolyzed into active caspase-1 (9, 22). ASC is composed of two death domains: an N-terminal PYD and a C-terminal CARD. The PYD domain can be linked to NLRP3, and CARD can recruit caspase-1 (23). Finally, pro-IL-1β was hydrolyzed into the mature and active IL-1β by caspase-1. Notably, caspase-1 exists in atherosclerotic plaques in humans (24). Sometimes, the production of IL-1β was not dependent on the hydrolytic process catalyzed by caspase-1. It could also be triggered by other enzymes such as neutrophil proteinase 3 (PR3) and matrix metalloprotease 9(MMP9) (25, 26).

### IL-1R Signal Transduction

**Figure 1:**
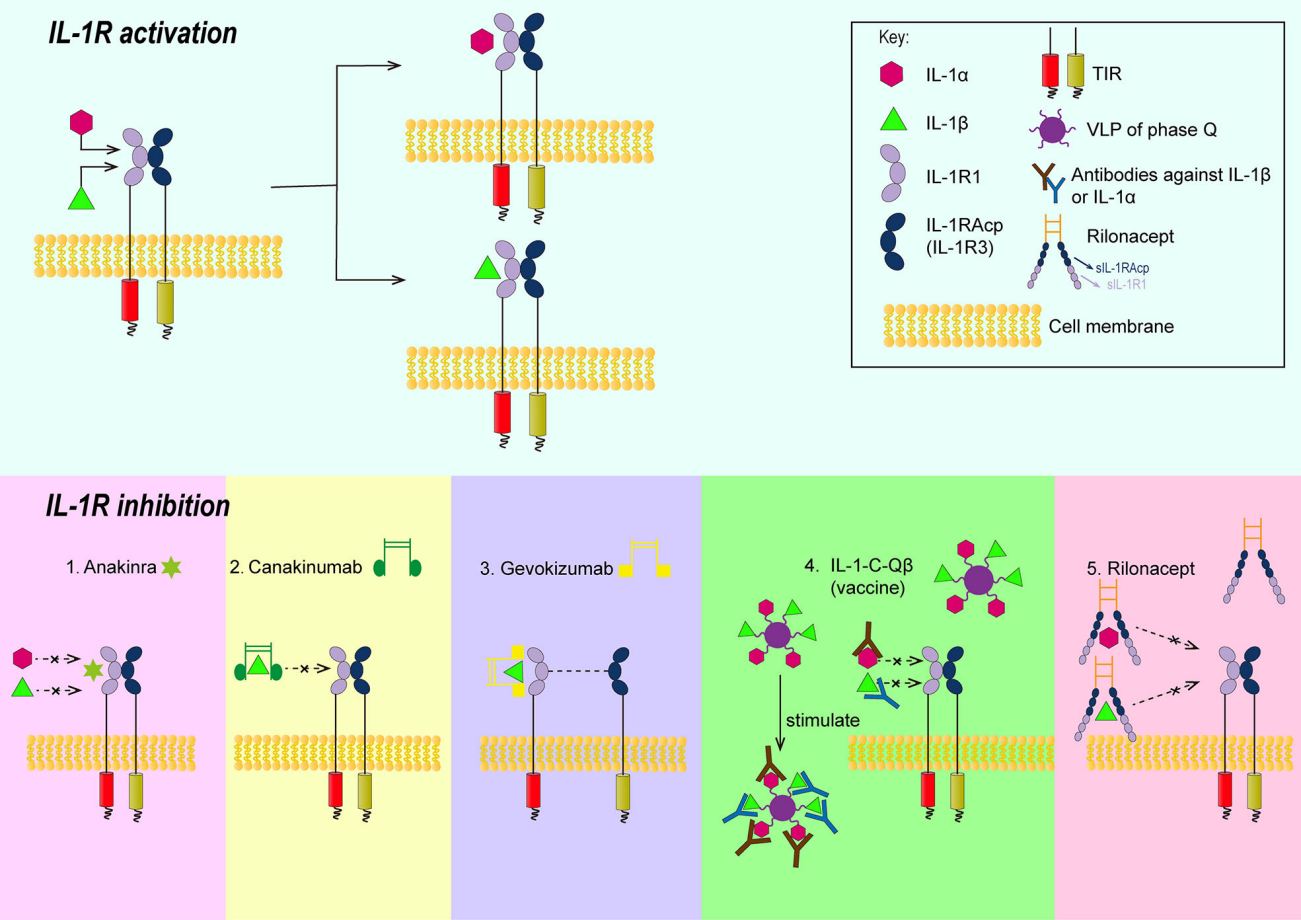
Schematic illustrations showing the approaches of controlling signal transduction of IL-1β. 1. Anakinra can competitively bind with IL-1R1 against IL-1β or IL-1α; 2. The Fab segment of canakinumab overlapped the D1 region of IL-1R1 when canakinumab bind to IL-1β, so that IL-1 βcould not bind to IL-1R1; 3. gevokizumab occupies the allosteric site of IL-1 and reduces the affinity of the complex to IL-1Racp and IL-1R1; 4. IL-1-C-Qβ(vaccine) produces polyclonal antibodies to neutralize IL-1; 5. Extracellular regions of IL-1R1 and IL-1Racp form rilonacept’s two arms that could trap IL-1. IL-1R, IL-1 receptor; TIR, the Toll-and IL-1R-like domains; VLP, virus like particles; IL-1R1, type II L-1R; IL-1 Racp, IL-1R accessory protein.

The process of IL-1R family signal transduction could also be divided into several steps and part of them were summarized in **Figure 1**. After the binding of IL-1 receptor with their ligands, IL-1β, IL-1R type I (IL-1R1) and IL-1R accessory protein (IL-1Racp) form heterotrimers (27). Meanwhile, the Toll- and IL-1R-like (TIR) domains existed on the receptor IL-1R1 and co-receptor IL-1Racp form heterodimers (28, 29). Oligomerized TIR recruits adaptor molecule, namely, myeloid differentiation primary response gene 88 (MyD88), to form a receptor complex with a high affinity, which can recruit downstream signaling molecules (30). The signal can be transducted *via* cytoplasmic kinase, like IL-1R associated kinase(IRAKs), and other adaptors, like TNF receptor associated factor 6 (TRAF 6) (31, 32). If the signal was transducted *via* the NF-κB pathway, the last step would be the phosphorylation of the inhibitor IκB by the IκB kinase, and the resultant release of the transcription factor NF-κB (33).

A series of phosphorylation and ubiquitination after the binding of IL-1β to IL-1R1 activate signal pathways such as NF-κB, JNK, p38 MAPK and induce expressions of genes, such as IL-6, IL-8, MCP-1, COX-2, IL-1α, IL-1β (34). Among them, NF-κB is an important one. Under normal conditions, the complex that consists of inhibiting protein IκBα and NF-κB heterodimer constituted by RelA and p50 exists in the cytoplasm. After the activation of inflammatory signals, IKB kinase (IKK) phosphorylates IκBα protein, leading to the ubiquitination of the latter, and the separation of IκBα from NF-κB. NF-κB is thus activated and translocates into the nucleus, then binds with specific DNA sequences. Next, the DNA/NF-κB complex recruits other proteins, and increases the transcription and translation of inflammatory mediator genes (35).

### The Effect of IL-1β on Atherosclerosis

IL-1β has multiple effects in all stages of atherosclerosis (12, 36). IL-1β induces an inflammatory response in endothelial cells, as reflected by increased expressions of adhesion factors and chemokines, and promotes the accumulation of inflammatory cells in blood vessels and their invasion into the local intima of blood vessels, which often happens at the initiation of atherosclerosis (37). In general, adhesion molecules include intercellular inflammatory cytokines (ICAM-1), vascular cell molecule (VCAM-1), chemokines include monocyte chemoattractant protein (MCP-1). Among them, MCP-1 could recruit mononuclear phagocytes and is closely related to atherosclerosis. IL-1 also stimulates the proliferation, differentiation of vascular smooth muscle cells, the activation of monocytes, macrophages and the secretion of various inflammatory mediators (38). IL-1β promotes the gene expression of a variety of inflammatory mediators: the enhanced expression of IL-1 itself forms a positive feedback loop; the induced cyclooxygenase-2 (COX-2) formation leads to production of prostaglandin; the generation of IL-6 and matrix metalloproteinase (MMP) can also be induced by IL-1β (39–41). IL-6 mediates the acute phase response, increasing the reactants in the acute phase, including C-reactive protein(CRP), fibrinogen and plasminogen activator inhibitors, which are closely related to the formation of atherosclerotic thrombosis (39–41). MMP1, MMP8, and MMP13 are also known as collagenase. They are closely related to the rupture of atherosclerotic fibrous cap plaque due to their characteristic of breaking down collagen (42). In addition, this cytokine can impair the contractility of myocardial cells and exacerbate post-infarction reperfusion injury (43). Notably, IL-1β plays a vital role in the growth of established atheroma. In experimental atherosclerosis, it has been proved that the selective neutralization of IL-1β promotes monocytes to switch to a less inflammatory state in plasma, elicits higher plasma level of IL-10, and lessens atherosclerosis plaque size without limitation of compensatory outward remodeling in the artery (44).

## Choice of IL-1α or L-1β to Target in Treating Atherosclerosis

IL-1α and IL-1β bind to the same receptor (IL-1R1) and therefore have similar downstream biological characteristics. However, they are different in terms of their cellular source, maturation requirements and release, which affect their impact on inflammation (45). Now that IL-1α and IL-1β both induce an inflammatory response by binding to IL-1R1, and have similar biological characteristics, what happens if IL-1R was both blocked? A study examining the effect of IL-1R1 deficiency on diet-induced atherosclerosis in ApoE^−/−^ mice showed a reduction in the size of atherosclerotic lesions with signs of plaque instability (46). Moreover, a Mendelian randomization analysis (human genetic data) suggests that genetic upregulation of the interleukin 1 receptor antagonist could increase cardiovascular risk in the long run (47). Therefore, we hypothesized that blocking both IL-1α and IL-1β would have more side effects than benefits.

As a member of the IL-1 family proteins, IL-1α is constitutively expressed by many non-immune cells and is also expressed by immune cells in an inflammatory environment (48). IL-1α precursors are released upon necrosis and injury of the cells that express IL-1α. Unlike IL-1β, IL-1α precursors also have pro-inflammatory activity. IL-1α is also associated with atherosclerosis (49–51). However, there are some negative evidence. In a small clinical trial, Xilonix, an IL-1α–targeted monoclonal antibody, was used in patients with femoral artery stenosis and was not significantly associated with a trend toward patency during three months of intravenous administration (52). In addition, IL-1α is the primary mediator of an IL-1R1–dependent and protective innate immune response against mycobacterium tuberculosis in mice; the mice with IL-1α block was more prone to infections than those with IL-1β block (53). Besides, antibodies against IL-1α impair outward vessel remodeling during early atherogenesis in Apoe^−/−^ mice fed a western diet, which could be a risk of plaque rupture in human. And the potential mechanism may well be that inhibition of IL-1α might decrease the production of MMP3 and SMC chemotaxis (44). Furthermore, atherosclerotic-associated inflammatory stimulation can induce inflammasomes that process IL-1β instead of IL-1α (12). Overall, IL-1α is a potential target for the treatment of atherosclerosis, although the target is not perfect.

## Inhibition of IL-1β and Randomized Clinical Trials

The role of IL-1β is tightly correlated with its signal transduction. Thus, inhibition of its signal transduction might be a way to alleviate atherosclerosis. There are several molecules that can inhibit the signal transduction of IL-1β (45): 1) IL-1Ra. IL-1Ra can competitively bind with IL-1R1 against IL-1β or IL-1α. The antagonist can tightly bind to IL-1R1 but is unable to recruit IL-1Racp (IL-1R3) to form TIR dimers. Thus it cannot mediate corresponding intracellular signaling. 2) IL-1R2. IL-1R2 is a decoy receptor that binds to IL-1α or IL-1β and can recruit IL-1R3, which is structurally similar to IL-1R1 but has no intracellular domain and was therefore unable to form TIR dimers. 3) Soluble receptors. Soluble receptors were also unable to mediate intracellular signaling, although there is no mature bait receptor or soluble receptor at present. In addition to them, there are animal experiments and clinical trials demonstrating drugs targeting IL-1β in the treatment of atherosclerosis. The drugs involve Anakinra, monoclonal antibodies, vaccines, rilonacept and were summarized in **Figure 1**.

### Endogenous Interleukin-1 Receptor Antagonist

Endogenous IL-1Ra is widely distributed and exhibits two forms, namely intracellular form and secretory form. The role of IL-1Ra in atherosclerosis is salutary in various animal experiments. In a study involving the overexpression or knockout of IL-1Ra in ldlr^−/−^ animal models, IL-1Ra knockout in C57BL/6J mice have been shown to increase plasma lipoprotein levels, when these animals were fed with a high-cholesterol/high-fat diet containing cholate; while a reduced size of lesions composed of foam cells was observed with the same chow (54). Similar results were observed in ApoE^−/−^ mice, as reflected by a significant increase of 30% of the atherosclerotic plaque in 16-week-old IL-1Ra+/^−^ mice fed with a normal diet compared with their IL-1Ra^−/−^ counterparts (55). Similar results were also observed in 32-week-old IL-1Ra+/^−^ mice with a tendency of plaque instability, as reflected by increased macrophage accumulation and decreased SMC content (55). Studies have shown that IL-1Ra knockout mice will develop lethal arterial inflammation with a large number of CD4+T cells, macrophages, and neutrophils infiltrating in the lesion area (56, 57). These results suggest that IL-1Ra plays an important role in inhibiting the development of lesions in the early stage of atherosclerosis, is involved in the regulation of plaque composition in the late stage, and is associated with complications such as plaque rupture and subsequent thrombosis. However, human genetic data is inconsistent with the above animal studies, which suggests that an inherited increase in IL-1Ra is associated with an increased cardiovascular risk (47). Moreover, the level of IL-1Ra is positively correlated with cardiovascular risk, although this is most likely the result of elevated IL-1 levels in an inflammatory state, which means that the improvement in IL-1Ra is the result rather than the cause (58). This issue is rather complicated and is needed more effort to expound.

### Recombinant IL-1 Receptor Antagonist

Anakinra is a recombinant human interleukin receptor antagonist that can antagonize IL-1α and IL-1β. It is usually used as a second-line therapy after the failure of diseases-modifying antirheumatic drug (DMARD) (59). It can also be used together with DMARD. Clinical trials have been carried out to investigate the effect of anakinra in acute coronary syndrome. In a phase II double-blind, randomized, placebo-controlled study enrolling 182 patients with non-ST-segment elevation acute coronary syndrome (NSTE-ACS) within 48 hours after onset of chest pain (60), daily subcutaneous injections of anakinra or placebo were administered for 14 days. During the 14 days’ treatment, CRP and IL-6 levels were significantly reduced in the anakinra group initially and returned to levels higher than those in the placebo group after 30 days. In another phase II/III, randomized, double-blind clinical trial named Virginia Commonwealth University Anakinra Remodeling Trial 3(VCUART3), anakinra was used in patients with ST-segment elevation myocardial infarction (STEMI) within 12 hours after the onset of chest pain (61). The primary endpoint of this trial was CRP levels 14 days after admission. Delightfully, in VCUART3, hsCRP, mortality, new-onset heart failure or death and hospitalization related to heart failure was significantly lower in anakinra-treated patients than in the placebo group during the 14 days of treatment. The results suggest that interleukin-1 blockers significantly reduced the acute inflammatory response in patients with ST-segment elevation myocardial infarction, and that the reduction in inflammatory signals was associated with a significant reduction in cardiovascular events. Anakinra well inhibits IL-1 signaling and thus alleviates the disease, but it is not suitable for chronic disease management due to its frequent usage. The emergence of monoclonal antibodies will solve this problem.

### Monoclonal Antibodies

Monoclonal antibodies (Canakinumab or Gevokizumab) can selectively target IL-1β without affecting other cytokines in the IL-1 family, reducing the risks of drug adverse effects, including infections. Canakinumab promotes the formation of an antigen-antibody complex by binding specifically to IL-1β. The complex prevents IL-1β from binding to IL-1R1 and thus blocks IL-1 signaling. This process is not directly related to IL-1Racp. By using Protein Data Bank (PDB), the structural data of IL-1R1 and IL-1β were used for modeling; it was found that after the binding of canakinumab to IL-1β, the Fab segment of the monoclonal antibody overlapped the D1 region of IL-1R1 significantly, so that IL-1β could not bind to the receptor again. Gevokizumab, another monoclonal antibody for IL-1β, is under active study. Unlike canakinumab, it occupies the allosteric site of IL-1 and reduces the affinity of the complex to IL-1Racp and IL-1R1 (62).The effect of gevokizumab has also been tested in animal studies which had a sound result (63, 64). It is worth mentioning that Gevokizumab is being used in cancer research and canakinumab was noticed to be salutary in cancer therapy in CANTOS.

Canakinumab has been approved for the treatment of cryopyrin-associated induction (CAPS) in 2009 by the U.S. Food and Drug Administration (FDA) (65). The antibody was approved in 2016 for three types of spontaneous inflammatory diseases: tumor necrosis factor receptor associated periodic syndrome (TRAPS), hyperimmunoglobulin D syndrome (HIDS)/mevalonate kinase (MKD), and familial Mediterranean fever (FMF) (66). It was also approved for active Still’s disease in 2020 (67). In terms of clinical results, CANTOS presented at the Annual Meeting of the European Association for Cardiology in 2017 finally corroborated the inflammation hypothesis. In this clinical trial, 10061 volunteers with CRP > 2 mg/L after previous second-line preventive treatment of myocardial infarction were randomly divided into a placebo group or canakinumab treatment groups of 50 mg/150 mg/300 mg dosage. The patients were injected subcutaneously once every quarter. Patients treated with 150 mg canakinumab had a 15% lower incidence of primary clinical end events, including non-fatal myocardial infarction, non-fatal stroke, and cardiovascular death, with somewhat increased infections, compared with those in the placebo group. In a pre-specified secondary analysis of trial results, the compound primary endpoint was reduced by 25%, while all-cause and cardiovascular mortality was reduced by 31% in patients treated with canakinumab (68). Even though the monoclonal antibody groups have higher incidence of fatal infection (incidence rate, 0.31 vs. 0.18 events per 100 person-years; P=0.02), there is no significance in all-cause mortality. This is most likely because the inflammatory symptoms are masked after IL-1 blockade and diagnosis is delayed.

Surprisingly, canakinumab reduced inflammation but led to the development of plaque instability in Apoe^−/−^ Mice (69). Although IL-1β appears to have a protective effect against atherosclerosis here, there are several issues that need be taken into account. First of all, there are often inconsistent results between animal experiments and clinical trials for targeted drugs, due to the inconsistency in the physiological and pathological process between animal models and human disease. In experiments involving non-high-fat or restrictive high-fat diet, it can often be proved that IL-1β signaling pathway could promote atherosclerosis (70). Second, it can be inferred from the earlier animal experiments on IL-1Ra that IL-1β plays a more important role in early stages of atherosclerosis, while the animal model here is in a late stage (55, 70, 71). Thirdly, in this experiment, macrophages were shown to polarize towards the M2 phenotype. In addition, when SMC-specific IL1r1 gene is knocked out in this experiment, the atherosclerotic plaque is reduced; it is unavoidable for various cells to be reduced during this process, and SMC reduction is not necessarily a bad sign. Thus, the role of IL-1β in atherosclerosis is still believed to be detrimental in anti-inflammatory treatment, in addition to CANTOS, other off-target therapies have also been tried. Take statins for example, which is typically used to reduce low-density lipoprotein cholesterol (LDL-C) levels and has been proved that consistently reduced vascular inflammation (72). Of note, the use of stains may promotes the NLRP3 and type I interferon pathways and both are considered to be proatherosclerotic (73, 74). Recent research show evidence that colchicine may reduce the cardiovascular risk in patients with chronic coronary disease (75), but the evidence is weak and the side effects is significant, especially in gastrointestinal system. Apart from that, compared with off-target therapies, specificity and lack of effect on lipids give targeting IL-1 therapies more specificities and less side effects.

### IL-1 Vaccines

IL-1 vaccine could be produced when IL-1 was transformed into a kind of antigen with a cysteine at the carboxyl end, and virus like particles(VLP) of phage Q was used as carriers. The carriers could be cross-linked with IL-1β or IL-1α to form IL-1β-C-VLP/IL-1β-C-Qβ or IL-1α-C-VLP/IL-1α-C-Qβ vaccines, respectively (76). This method can overcome the immune tolerance to autoantigens without adjuvant usage, which is a major difference compared to previous vaccine-producing methods (77, 78). The polyclonal antibodies produced can block the binding of IL-1β to IL-1R1. Currently, such vaccines have been used in several animal models of cardiovascular disease (79, 80). The advantage of vaccines is their inexpensiveness compared to monoclonal antibodies, which might lead to better patient compliance.

### IL-1 Inhibitor

In addition to the abovementioned molecules, IL-1 inhibitor might also be a potential treatment method for atherosclerosis. Rilonacept, also known as IL-1 trap, is an IL-1 inhibitor. It is an antibodily-like dimer protein with two arms composed of the ligand-binding regions on the extracellular regions of IL-1R1 and IL-1Racp. The two arms are linked by Fc segments and can neutralize IL-1 (81).

## Summary

Countless researchers have made great efforts in the investigations of cardiovascular inflammation and there are abundant lessons that we can learn from them. First of all, better simulation of human disease characteristics in animal models requires our joint efforts. Second, different stages of atherosclerosis have different characteristics, only a grasp of their different characteristics can lead to a better treatment. Thirdly, from the perspective of the failed clinical trials, considering hsCRP as an inclusion criterion for anti-inflammatory therapy may lead to a better effect observed in anti-inflammatory therapy, and regarding FDG-PET as a quantitative indicator of vascular inflammation may result in a better observation of the vasculature. Hence, finding more effective markers of inflammation is also something we need to work on. In addition, lipid-lowering and anti-inflammatory therapies are not antithetical to each other, and that the best use of these two weapons will lead to a brighter future for the treatment of atherosclerosis. Finally, bioinformatics is booming recently, however, the room for improvement in applying bioinformatic knowledge in cardiovascular field is much larger compared with that in oncology. Nevertheless, efforts are still needed to develop cardiovascular proprietary bioinformatics databases.

## Author Contributions

All authors listed have made a substantial, direct, and intellectual contribution to the work and approved it for publication.

## Conflict of Interest

The authors declare that the research was conducted in the absence of any commercial or financial relationships that could be construed as a potential conflict of interest.
